# An inflammatory–nutritional machine learning model for risk stratification of hospital-acquired pneumonia in traumatic brain injury: a multicenter study

**DOI:** 10.3389/fnut.2026.1785139

**Published:** 2026-05-28

**Authors:** Chenzhu Cai, Zhenyu Fan, Longjie Chen, Shanglu Lin, Mingfa Cai, Zhen Qi, Xieli Guo

**Affiliations:** 1 Department of Neurosurgery, Jinjiang Municipal Hospital (Shanghai Sixth People’s Hospital Fujian), Quanzhou, China; 2 School of Software, North University of China, Taiyuan, China; 3 Department of Neurosurgery, Second Affiliated Hospital of Fujian Medical University, Quanzhou, China

**Keywords:** functional prognosis, hospital-acquired pneumonia, machine learning, Pan-immune-inflammation value, prognostic nutritional index, traumatic brain injury

## Abstract

**Background:**

Hospital-acquired pneumonia (HAP) is a frequent and serious complication following traumatic brain injury (TBI), leading to prolonged hospitalization and poor functional outcomes. Early identification of patients at high risk of HAP remains challenging. Systemic inflammation and nutritional status are recognized contributors to post-TBI infection susceptibility; however, these factors are not adequately incorporated into existing predictive models. This study aimed to develop and validate an inflammatory–nutritional machine learning model for predicting HAP after TBI and to evaluate its prognostic stratification performance using an independent testing cohort from a second center.

**Methods:**

A total of 567 adult patients with TBI were included in this retrospective multicenter cohort study conducted at two hospitals. Patients were divided into a training set (n = 396) and an independent testing set (*n* = 171). Baseline laboratory data obtained within 24 h of admission were used to calculate the Pan-Immune-Inflammation Value (PIV) and Prognostic Nutritional Index (PNI) scores. HAP, defined as occurring ≥48 h after admission, was designated as the primary outcome, and functional prognosis at discharge was assessed using the modified Rankin Scale (mRS). Six machine learning models were constructed and compared. Model performance was evaluated using discrimination, calibration, decision curve analysis, and 10-fold cross-validation. Model interpretability was assessed with Shapley Additive Explanations (SHAP), and Kaplan–Meier analyses were conducted for prognostic stratification.

**Results:**

The Light Gradient Boosting Machine exhibited the best overall performance, achieving an area under the receiver operating characteristic curve of 0.815 in the testing cohort, with good calibration and superior clinical net benefit. Cross-validation confirmed stable predictive capability. SHAP analysis identified PIV as the most influential predictor, followed by PNI, demonstrating consistent feature importance across cohorts. Model-derived risk stratification was significantly associated with functional outcomes, with high-risk patients exhibiting a markedly lower likelihood of favorable prognosis (mRS 0–2) in both cohorts.

**Conclusion:**

The inflammatory–nutritional machine learning model integrating PIV and PNI provides accurate and interpretable prediction of HAP after TBI and effectively stratifies functional prognosis, supporting its potential value for early risk assessment and future individualized decision-support in patients with TBI, pending prospective validation.

## Background

Traumatic brain injury (TBI) is a leading cause of morbidity and mortality worldwide and is frequently complicated by hospital-acquired pneumonia (HAP), one of the most common and severe secondary complications during hospitalization (1, 2). Patients with TBI are particularly susceptible to HAP because of impaired consciousness, dysphagia, prolonged immobilization, mechanical ventilation (3), and TBI-induced immune dysregulation (4). The development of HAP is associated with extended hospital stay, increased healthcare burden, and poor neurological and functional outcomes. Therefore, early identification of patients at high risk for HAP is essential for timely prevention and optimized clinical management.

Systemic inflammation and nutritional status play central roles in vulnerability to post-TBI infection. Acute brain injury triggers profound immune dysregulation, characterized by excessive activation of innate immunity, lymphocyte suppression, and platelet–monocyte interactions, all of which contribute to increased infection susceptibility (5). Concurrently, malnutrition is common after severe neurological injury and is independently associated with impaired immune defense, delayed recovery, and increased infection risk (6, 7). Despite this biological significance, most existing HAP prediction models for TBI populations rely mainly on demographic or injury-related variables, with limited integration of objective inflammatory or nutritional biomarkers.

Composite indices may more effectively capture the complex immune–nutritional milieu following TBI. The Pan-Immune-Inflammation Value (PIV), which integrates neutrophil, lymphocyte, platelet, and monocyte counts, provides a comprehensive evaluation of systemic inflammatory and immune status (8); however, its predictive role in post-TBI HAP has not been fully investigated. Similarly, the Prognostic Nutritional Index (PNI), which combines serum albumin level and lymphocyte count, reflects the nutritional–immune reserve but is rarely incorporated into neurotrauma-related infection models (9). Furthermore, previous studies have often been limited by single-center design, lack of independent center-based validation, and reliance on traditional regression approaches, while the prognostic implications of HAP risk stratification for functional outcomes remain insufficiently examined. Therefore, this two-center study aimed to develop and validate using an independent testing cohort from a second center. An inflammatory–nutritional machine learning model incorporating PIV and PNI to predict HAP after TBI and to evaluate its utility for functional prognostic stratification.

## Methods and materials

### Study population and patient selection

This was a retrospective two-centers cohort study. All the patients were consecutively enrolled from two tertiary medical centers during predefined study periods: Jinjiang Municipal Hospital (January 1, 2021 to December 31, 2025) and the Second Affiliated Hospital of Fujian Medical University (January 1, 2023 to December 31, 2025). Patients with TBI who met the predefined eligibility criteria. The inclusion criteria were as follows: (1) Age ≥18 years at admission; (2) Confirmed diagnosis of TBI based on clinical evaluation and neuroimaging (computed tomography or magnetic resonance imaging); (3) Hospital admission within 24 h after injury; (4) Availability of baseline laboratory data required for calculating PIV and PNI; and (5) Complete in-hospital follow-up data, including HAP status and functional outcomes at discharge assessed using the modified Rankin Scale (mRS).

Patients were excluded if they met any of the following criteria: (1) Pre-existing pneumonia or pulmonary infection at admission, or pneumonia developing within 48 h after admission; (2) History of chronic inflammatory, autoimmune, hematologic, or malignant disease; (3) Severe hepatic or renal dysfunction at baseline, including liver failure or end-stage renal disease requiring dialysis; (4) Use of immunosuppressive therapy before admission; (5) Missing or incomplete key clinical or laboratory data; (6) In-hospital death or discharge within 48 h; or (7) Repeat admissions or transferred cases with unverifiable baseline data or outcomes.

After applying these criteria, eligible patients from Jinjiang Municipal Hospital were assigned to the training set for model development, whereas patients from the Second Affiliated Hospital of Fujian Medical University were assigned to an independent testing set for center-based validation.

### Treatment methods

All patients received timely and appropriate management according to standard clinical practice guidelines for traumatic brain injury at Jinjiang Municipal Hospital and the Second Affiliated Hospital of Fujian Medical University. Treatment strategies, including conservative medical management and surgical interventions (such as decompressive craniectomy or hematoma evacuation when indicated), were determined by experienced neurosurgeons based on neurological status, neuroimaging findings, and intracranial pressure evaluation. All therapeutic decisions followed institutional protocols and standard care procedures at both medical centers.

### Collection and definition of predictor and outcome variables

Baseline demographic characteristics, clinical data, and laboratory parameters were retrospectively extracted from the electronic medical record systems of the participating centers. Laboratory indices were obtained from peripheral venous blood samples collected within 24 h of admission, prior to the onset of HAP. The primary predictor variables were PIV and PNI. PNI was calculated as serum albumin concentration (g/L) plus five times the peripheral lymphocyte count (×10^9^/L), representing baseline nutritional and immune status (10). PIV was calculated as the product of neutrophil, platelet, and monocyte counts divided by the lymphocyte count (all ×10^9^/L), providing a composite indicator of systemic inflammatory and immune response (11). All cell counts were derived from the same complete blood count with differential, and serum albumin levels were measured using standard biochemical assays. The restriction of the primary model to PIV and PNI was prespecified and hypothesis-driven, because these two indices capture two biologically complementary dimensions of post-TBI infection susceptibility—systemic inflammatory and immune dysregulation, and nutritional–immune reserve—while remaining readily obtainable from routine admission laboratory tests without additional cost or subjective clinical assessment (4, 6, 12, 13).

The primary outcome was HAP, defined as pneumonia developing ≥48 h after admission and absent at the time of admission. Diagnosis required new or progressive pulmonary infiltrates on chest imaging accompanied by at least two clinical criteria: abnormal body temperature, leukocytosis or leukopenia, purulent respiratory secretions, or worsening oxygenation. Diagnoses were confirmed through physician documentation and radiological reports, with microbiological findings reviewed when available (13). The same predefined HAP diagnostic criteria were applied across both participating centers during retrospective data extraction and outcome ascertainment to improve cross-center consistency. For that, there was no formal inter-rater reliability assessment or central adjudication process was available. The secondary outcome was functional prognosis at discharge, assessed using the mRS. For analysis, mRS scores were dichotomized as favorable (0–2) or unfavorable (3–6), based on standardized neurological evaluations documented in the medical records (14).

### Ethical statement

This retrospective multicenter observational study was conducted in accordance with the principles of the Declaration of Helsinki. The study protocol was reviewed and approved by the Institutional Ethics Committees of Jinjiang Municipal Hospital and the Second Affiliated Hospital of Fujian Medical University. Owing to the retrospective design and the use of anonymized data obtained during routine clinical care, the requirement for written informed consent was waived by the respective ethics committees. All patient data were de-identified before analysis to ensure confidentiality and data security.

### Statistical analysis

All statistical analyses and model development were performed using Python version 3.12. Data processing and descriptive analyses were conducted with NumPy and Pandas. Group comparisons were carried out with SciPy, applying the Student’s t test or Mann–Whitney U test for continuous variables, and the χ^2^ test or Fisher’s exact test for categorical variables, as appropriate. A two-sided *p* value < 0.05 was considered statistically significant.

Participants with missing or incomplete key clinical or laboratory variables were excluded according to the predefined eligibility criteria. Among the 786 patients initially screened, 219 (27.9%) were excluded for this reason; therefore, the final analyses were conducted using a complete-case dataset of 567 patients, and no imputation procedure was applied. The predictive models were based on two prespecified continuous variables, namely the PIV and the PNI, and no additional feature selection procedure was performed. All models were fitted using the original continuous values of PIV and PNI, and no feature scaling or standardization procedure was applied.

Machine learning models, including logistic regression, decision tree, random forest, support vector machine, extreme gradient boosting (XGB), and Light Gradient Boosting Machine (LGBM), were implemented using scikit-learn, XGBoost, and LightGBM. To address class imbalance in the training cohort, the Synthetic Minority Over-sampling Technique (SMOTE) was applied during model development. For 10-fold cross-validation within the training cohort, SMOTE was applied separately within each training fold, while the corresponding validation fold remained unresampled. The independent testing cohort was not resampled and remained in its original distribution for independent center-based evaluation. In the primary analysis, the default parameter settings of the corresponding Python packages were used for all machine learning models. In addition, a supplementary hyperparameter tuning analysis was performed for the LGBM model using randomized search with 10-fold cross-validation in the training set (Supplementary Table S3). Model performance was evaluated using the area under the receiver operating characteristic curve (AUROC), accuracy, recall, precision, F1-score, Matthews correlation coefficient (MCC), and specificity. Calibration curves, precision–recall curves, and decision curve analyses were applied to assess model calibration and potential clinical relevance. Internal validation of the optimal model was performed with 10-fold cross-validation. Model interpretability was evaluated using Shapley Additive Explanations (SHAP), with feature importance quantified by the mean absolute SHAP values in both the training and testing cohorts. Kaplan–Meier (K-M) analyses to visualize in-hospital separation in favorable functional outcome across model-defined risk groups during hospitalization. All figures were created using Matplotlib and Seaborn.

In addition, sensitivity analyses were performed in the training cohort to examine the robustness of the association between the model-derived risk estimate and HAP after adjustment for available baseline covariates. Three regression models were constructed: Model 1 was unadjusted; Model 2 was adjusted for Gender, Age, Hypertension, Diabetes Mellitus, Smoking, and Drinking; and Model 3 was further adjusted for GCS and WBC. Variables that were unavailable at admission or were constituent components of PIV and PNI were not included in the adjustment models.

## Results

### Patient selection and study workflow

**Figure 1:**
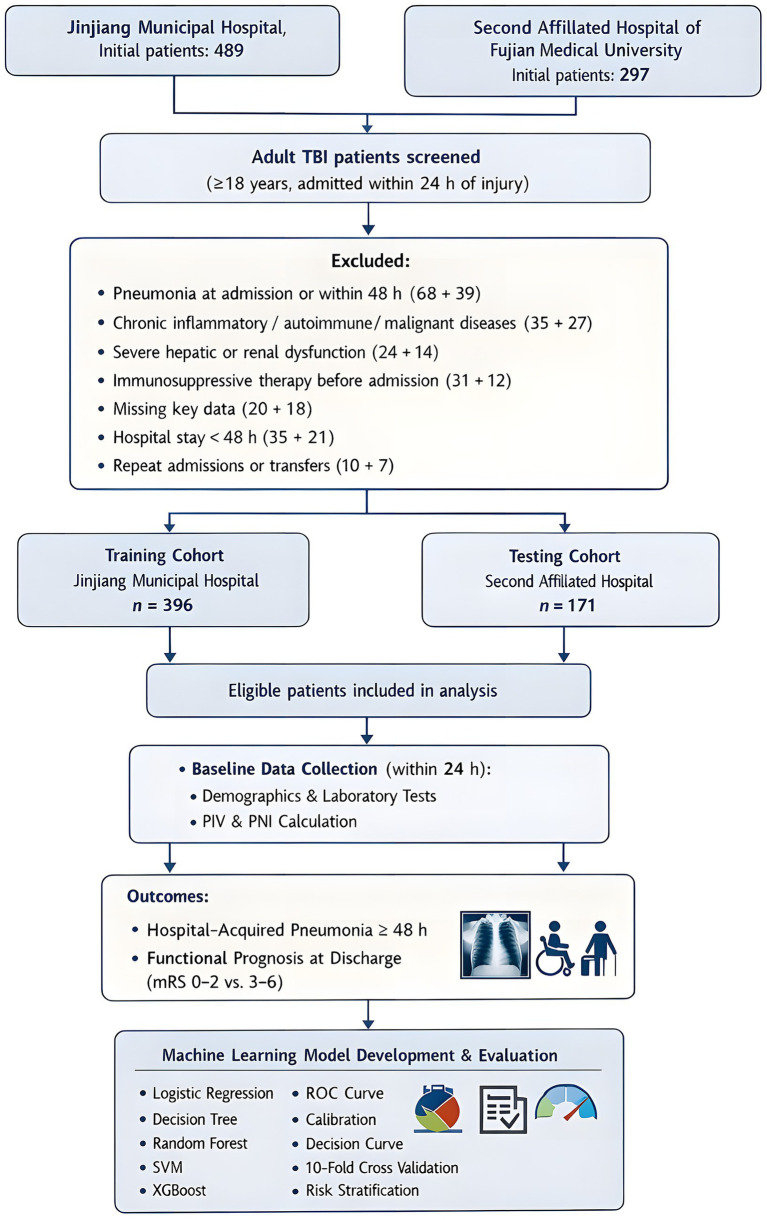
Patient selection and study workflow in a two-center predictive modeling study. This figure presents the patient selection process and study workflow of this two-center retrospective study. A total of 489 patients with TBI from Jinjiang Municipal Hospital and 297 from the Second Affiliated Hospital of Fujian Medical University were initially screened. After applying predefined inclusion and exclusion criteria, 396 patients were included as the training cohort and 171 as the independent testing cohort. Baseline data were collected to calculate the PIV and PNI. Hospital-acquired pneumonia was defined as the primary outcome, and functional prognosis at discharge was assessed using the mRS.

Figure 1 presents the patient selection process and overall study workflow. A total of 786 patients with TBI were initially screened across two medical centers. Specifically, 489 patients were identified from Jinjiang Municipal Hospital between January 1, 2021, and December 31, 2025, and 297 patients were identified from the Second Affiliated Hospital of Fujian Medical University between January 1, 2023, and December 31, 2025. After applying predefined inclusion and exclusion criteria, 396 patients were enrolled in the training set, and 171 eligible patients were assigned to the independent testing set.

After cohort derivation, baseline demographic, clinical, and laboratory data were collected within 24 h of admission. Key inflammatory–nutritional predictors, including PIV and PNI, were calculated for subsequent analyses. HAP was defined as the primary outcome, and functional prognosis at discharge, assessed using the mRS, served as the secondary outcome. This structured workflow facilitated model development in the training cohort and independent center-based validation in the testing cohort, ensuring robust evaluation of predictive performance and clinical applicability.

### Baseline demographic and clinical characteristics

In this study, patients were collected into training and testing set at a 7:3 ratio. Baseline demographic, clinical, and laboratory characteristics of both cohorts are summarized in Table 1. In the training set, patients who developed HAP were comparable to those without HAP in terms of age, sex, drinking history, and time from injury to admission (all *p* > 0.05). However, several comorbidities and injury-related variables differed significantly. Hypertension, diabetes mellitus, and smoking history were more prevalent among patients with HAP (all *p* < 0.001), who also exhibited lower admission Glasgow Coma Scale (GCS) scores (p < 0.001). Laboratory findings revealed lower serum sodium (*p* = 0.044) and albumin levels (*p* = 0.009), along with higher white blood cell, neutrophil, monocyte, and platelet counts (all *p* < 0.05), indicating an enhanced inflammatory response. Consistently, PIV was significantly higher in the HAP group (*p* < 0.001), whereas PNI did not differ significantly (*p* = 0.07). Clinically, HAP was associated with longer hospital stay, increased need for surgical intervention, and a higher rate of unfavorable functional outcomes (all *p* < 0.001).

**Table 1:** Baseline demographic and clinical characteristics.

Variable	Training set			*p*-value	Testing set			*p*-value
	Overall	Non-HAP	HAP		Overall	Non-HAP	HAP	
	*N* = 396	*N* = 241	*N* = 155		*N* = 171	*N* = 107	*N* = 64	
Age	57.65 ± 10.83	57.25 ± 10.19	58.28 ± 11.78	0.374	57.32 ± 11.17	56.32 ± 9.99	59.00 ± 12.82	0.154
Gender				0.521				0.026
Male	263.00 (66.41%)	163.00 (67.63%)	100.00 (64.52%)		109.00 (63.74%)	75.00 (70.09%)	34.00 (53.13%)	
Female	133.00 (33.59%)	78.00 (32.37%)	55.00 (35.48%)		62.00 (36.26%)	32.00 (29.91%)	30.00 (46.88%)	
HP				<0.001				0.007
No	130.00 (32.83%)	101.00 (41.91%)	29.00 (18.71%)		59.00 (34.50%)	45.00 (42.06%)	14.00 (21.88%)	
Yes	266.00 (67.17%)	140.00 (58.09%)	126.00 (81.29%)		112.00 (65.50%)	62.00 (57.94%)	50.00 (78.13%)	
DM				<0.001				0.146
No	357.00 (90.15%)	227.00 (94.19%)	130.00 (83.87%)		152.00 (88.89%)	98.00 (91.59%)	54.00 (84.38%)	
Yes	39.00 (9.85%)	14.00 (5.81%)	25.00 (16.13%)		19.00 (11.11%)	9.00 (8.41%)	10.00 (15.63%)	
Smoking				<0.001				0.042
No	342.00 (86.36%)	230.00 (95.44%)	112.00 (72.26%)		148.00 (86.55%)	97.00 (90.65%)	51.00 (79.69%)	
Yes	54.00 (13.64%)	11.00 (4.56%)	43.00 (27.74%)		23.00 (13.45%)	10.00 (9.35%)	13.00 (20.31%)	
Drinking				0.205				0.014
No	366.00 (92.42%)	226.00 (93.78%)	140.00 (90.32%)		158.00 (92.40%)	103.00 (96.26%)	55.00 (85.94%)	
Yes	30.00 (7.58%)	15.00 (6.22%)	15.00 (9.68%)		13.00 (7.60%)	4.00 (3.74%)	9.00 (14.06%)	
Time	5.59 ± 6.13	5.62 ± 5.91	5.55 ± 6.49	0.922	5.42 ± 4.73	5.62 ± 5.06	5.09 ± 4.14	0.464
GCS	10.35 ± 3.34	11.57 ± 2.88	8.45 ± 3.12	<0.001	10.33 ± 3.32	11.48 ± 2.80	8.41 ± 3.26	<0.001
K	3.54 ± 0.48	3.54 ± 0.44	3.54 ± 0.54	0.951	3.53 ± 0.45	3.59 ± 0.43	3.44 ± 0.48	0.038
Na	138.20 ± 3.52	138.49 ± 3.31	137.74 ± 3.79	0.044	138.08 ± 3.98	138.37 ± 3.72	137.60 ± 4.36	0.245
Ca	2.33 ± 0.22	2.35 ± 0.23	2.32 ± 0.20	0.188	2.32 ± 0.20	2.36 ± 0.20	2.26 ± 0.17	<0.001
GLU	10.65 ± 46.71	7.78 ± 2.64	15.12 ± 74.51	0.222	8.24 ± 2.94	7.93 ± 2.95	8.77 ± 2.86	0.069
Albumin	40.46 ± 4.70	41.00 ± 3.78	39.63 ± 5.76	0.009	40.60 ± 4.45	41.42 ± 3.66	39.24 ± 5.27	0.004
WBC	10.33 ± 4.15	9.09 ± 3.43	12.26 ± 4.43	<0.001	10.09 ± 3.58	9.17 ± 2.79	11.62 ± 4.21	<0.001
Neutrophile	8.43 ± 4.09	7.34 ± 3.34	10.12 ± 4.56	<0.001	8.18 ± 3.65	7.35 ± 2.87	9.58 ± 4.36	<0.001
Lymphocyte	1.35 ± 0.85	1.27 ± 0.56	1.48 ± 1.15	0.036	1.33 ± 0.85	1.28 ± 0.59	1.42 ± 1.17	0.361
Monocyte	0.49 ± 0.27	0.42 ± 0.21	0.60 ± 0.31	<0.001	0.49 ± 0.43	0.40 ± 0.17	0.64 ± 0.63	0.003
Hb	149.47 ± 21.77	151.37 ± 16.65	146.52 ± 27.74	0.051	147.61 ± 20.31	151.30 ± 14.48	141.44 ± 26.42	0.007
PLT	188.09 ± 67.79	181.37 ± 61.32	198.52 ± 75.81	0.019	205.28 ± 74.64	197.09 ± 55.27	218.97 ± 97.92	0.105
LOS	19.20 ± 13.87	14.39 ± 7.68	26.69 ± 17.57	<0.001	20.98 ± 15.85	14.79 ± 7.62	31.34 ± 20.16	<0.001
Surgery				<0.001				<0.001
No	244.00 (61.62%)	186.00 (77.18%)	58.00 (37.42%)		115.00 (67.25%)	86.00 (80.37%)	29.00 (45.31%)	
Yes	152.00 (38.38%)	55.00 (22.82%)	97.00 (62.58%)		56.00 (32.75%)	21.00 (19.63%)	35.00 (54.69%)	
mRS group (*n*%)				<0.001				<0.001
Favorable	241.00 (60.86%)	191.00 (79.25%)	50.00 (32.26%)		104.00 (60.82%)	85.00 (79.44%)	19.00 (29.69%)	
Unfavorable	155.00 (39.14%)	50.00 (20.75%)	105.00 (67.74%)		67.00 (39.18%)	22.00 (20.56%)	45.00 (70.31%)	
PIV	807.71 ± 900.41	575.85 ± 699.42	1,168.21 ± 1,049.86	<0.001	807.18 ± 759.91	564.30 ± 511.98	1,213.26 ± 921.68	<0.001
PNI	10.81 ± 4.25	10.45 ± 2.83	11.36 ± 5.78	0.07	10.71 ± 4.22	10.52 ± 2.86	11.03 ± 5.84	0.523

HAP, hospital -acquired pneumonia; TBI, traumatic brain injury; HP, hypertension; DM, diabetes mellitus; Time, time from injury onset to hospital admission; GCS, glasgow coma scale; K, serum potassium; Na, serum sodium; Ca, serum calcium; GLU, blood glucose; WBC, white blood cell count; Hb, hemoglobin; PLT, platelet count; LOS, length of hospital stay; mRS, modified rankin.

Scale, PIV, Pan-immune-inflammation value; PNI, prognostic nutritional index.

Similar patterns were observed in the testing set. Although age and most metabolic parameters were comparable, patients with HAP had higher rates of hypertension (*p* = 0.007), smoking (*p* = 0.042), and alcohol consumption (*p* = 0.014), along with lower admission GCS scores (*p* < 0.001). Inflammatory markers—including white blood cell, neutrophil, and monocyte counts—were consistently elevated (all *p* < 0.01), accompanied by a markedly higher PIV (*p* < 0.001). Serum albumin levels were significantly decreased (*p* = 0.004), whereas PNI again showed no significant difference (*p* = 0.523). Patients who developed HAP also had longer hospital stays, higher rates of surgical intervention, and poorer functional outcomes (all *p* < 0.001), confirming the consistency of these findings across cohorts.

### Model construction based on dual inflammatory–nutritional indices

The predictive model was developed by integrating PIV and PNI as continuous variables, representing the combined influence of systemic inflammation and nutritional–immune status on HAP risk after TBI. As shown in the baseline characteristics (Table 1), patients who developed HAP exhibited significantly higher PIV values in both the training and testing sets (*p* < 0.001), indicating marked inflammatory activation and immune dysregulation. Although PNI alone did not show statistically significant between-group differences in all subsets (e.g., *p* = 0.07 in the training cohort), it was retained because of its recognized importance in evaluating nutritional reserve and immunocompetence, which are critical for host defense against infection (15). Importantly, predictor selection in the present study was hypothesis-driven rather than based solely on univariate significance, and PNI was retained because it provides complementary biological information to PIV within the inflammatory–nutritional framework. By jointly incorporating PIV, which reflects inflammatory intensity, and PNI, which represents metabolic and immune capacity, the model provides a parsimonious inflammatory–nutritional assessment of patient susceptibility beyond either index alone, but it is not intended to represent a complete predictive model of all HAP risk determinants. This dual inflammatory–nutritional approach aligns with the immunonutrition framework of the present study and enhances the model’s potential clinical relevance for early risk stratification in patients with TBI.

### Overall model performance, calibration, and risk stratification across training and testing sets

**Figure 2:**
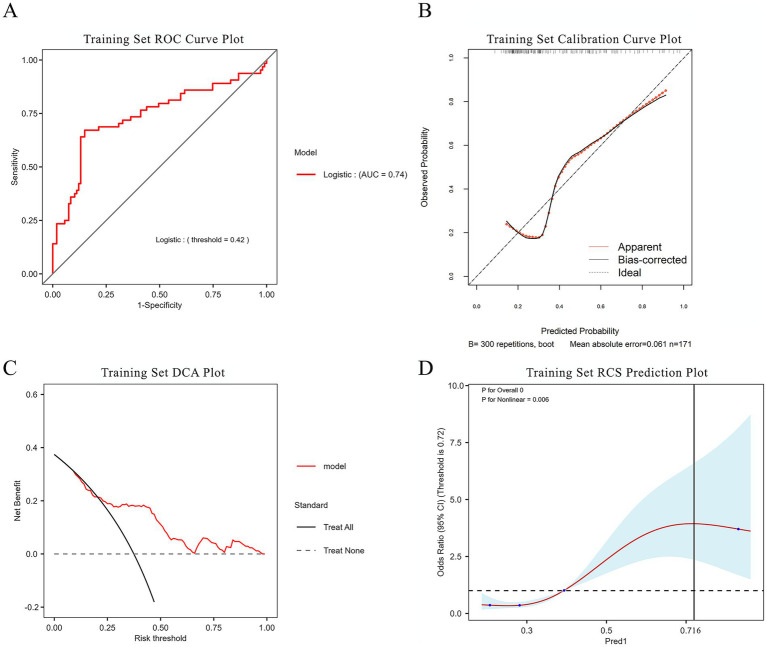
Discrimination, calibration, potential clinical relevance, and risk structure of the HAP predicted model in the training set. **(A)** The receiver operating characteristic (ROC) curve demonstrates the discriminative ability of the logistic regression model, with an area under the curve (AUC) of 0.74, indicating good overall discrimination between patients who developed HAP and those who did not. The diagonal gray line represents the reference line of no discrimination, and the optimal probability threshold is indicated on the plot. **(B)** The calibration curve depicts the agreement between predicted probabilities and observed HAP outcomes. The dashed diagonal line indicates ideal calibration, while the apparent (red) and bias-corrected (black) curves derived from 300 bootstrap resamples show close alignment with the ideal line across most risk ranges. The mean absolute error was 0.061, suggesting satisfactory calibration accuracy. **(C)** Decision curve analysis (DCA) evaluates the clinical usefulness of the model by quantifying net benefit across a range of threshold probabilities. The model (red line) provides a higher net benefit than both the “treat-all” and “treat-none” strategies over a broad interval of clinically relevant thresholds, supporting its potential value for guiding individualized clinical decision-making. **(D)** Restricted cubic spline (RCS) analysis illustrates the nonlinear association between the model-predicted risk (Pred1: the model prediction value) and the odds of developing HAP. The solid red line represents adjusted odds ratios, with the shaded area indicating 95% confidence intervals. A statistically significant nonlinear relationship was observed (*P* for nonlinearity = 0.006), demonstrating a progressively increasing risk of HAP with higher predicted probabilities. The vertical line denotes the selected risk threshold, and the horizontal dashed line indicates an odds ratio of 1.0.

Across the training and testing sets, the HAP prediction model demonstrated stable discrimination, reliable calibration, and substantial potential clinical relevance. In the training cohort, receiver operating characteristic (ROC) curve analysis yielded an area under the curve (AUC) of 0.74, indicating good discriminative capacity to differentiate patients who developed HAP from those who did not (Figure 2A). Calibration analysis showed close agreement between predicted and observed probabilities across most risk ranges, with both apparent and bootstrap bias–corrected curves closely following the ideal reference line and exhibiting a low mean absolute calibration error (Figure 2B). Decision curve analysis (DCA) further showed that the model offered greater net benefit than the “treat-all” and “treat-none” strategies across a wide range of threshold probabilities (Figure 2C). Restricted cubic spline (RCS) analysis revealed a significant nonlinear association between model-predicted risk and HAP occurrence (*P* for nonlinearity = 0.006), with progressively increasing risk at higher predicted probabilities (Figure 2D).

**Figure 3:**
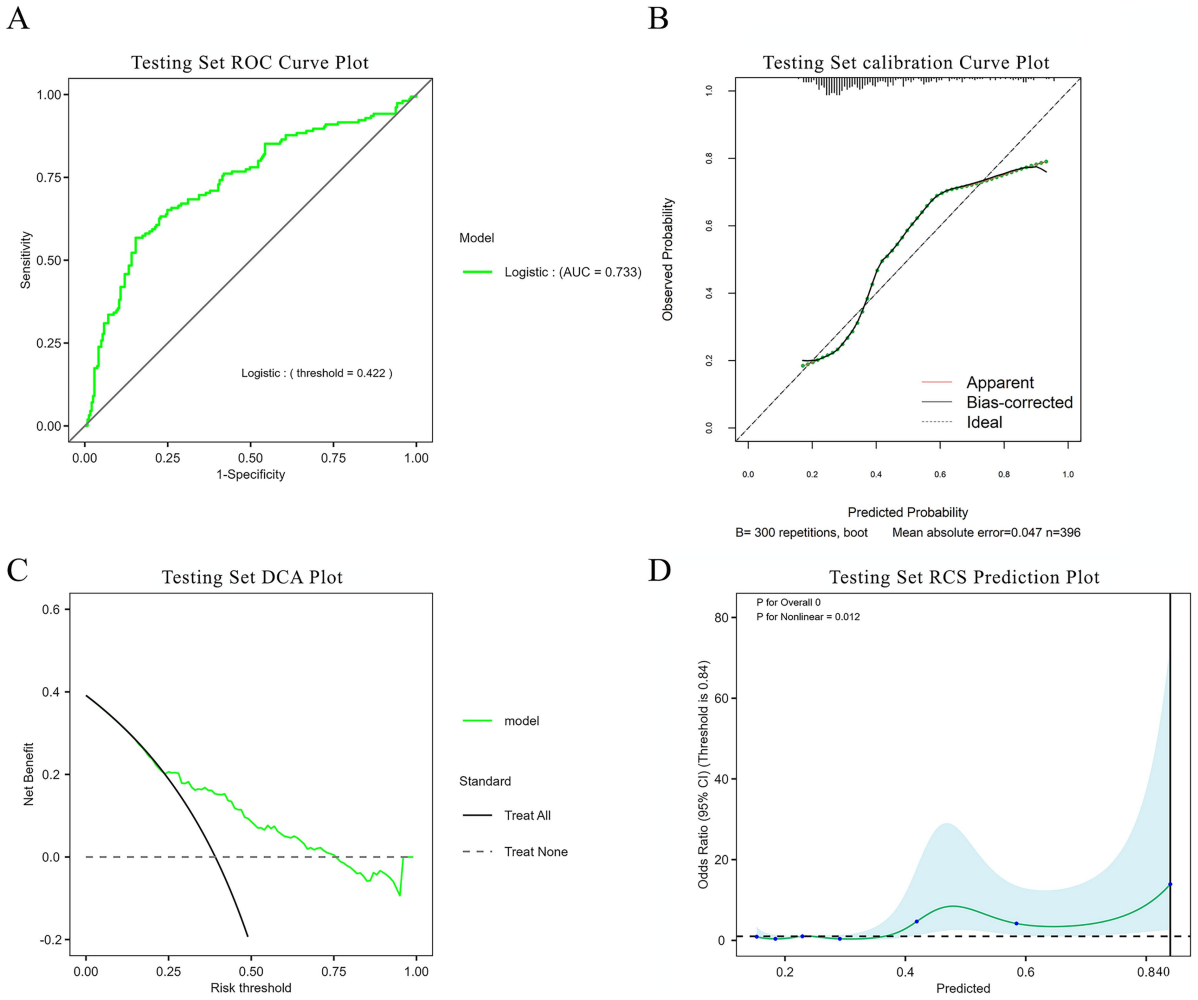
Discrimination, calibration, potential clinical relevance, and risk structure of the HAP prediction model in the testing set. **(A)** Receiver operating characteristic (ROC) curve demonstrating the discriminative ability of the model in the testing cohort, with an area under the curve (AUC) of 0.733, indicating consistent discrimination compared with the training cohort. The diagonal gray line represents the reference line of no discrimination, and the selected probability threshold is indicated on the plot. **(B)** Calibration curve comparing predicted probabilities with observed HAP outcomes in the testing set. The dashed diagonal line represents ideal calibration, while the apparent (green) and bias-corrected (black) curves derived from 300 bootstrap resamples demonstrate good agreement across most predicted risk ranges. The mean absolute calibration error was 0.047, indicating satisfactory calibration accuracy. **(C)** Decision curve analysis (DCA) assessing the clinical usefulness of the model. The model (green line) provides a higher net benefit than both the “treat-all” and “treat-none” strategies across a broad range of threshold probabilities, supporting its applicability for clinical risk stratification in the testing cohort. **(D)** Restricted cubic spline (RCS) analysis illustrating the nonlinear relationship between model-predicted risk and the odds of developing HAP. The solid green line represents adjusted odds ratios, with shaded areas indicating 95% confidence intervals. A statistically significant nonlinear association was observed (*P* for nonlinearity = 0.012), confirming a progressively increased risk of HAP at higher predicted probabilities. The vertical line denotes the selected risk threshold, and the horizontal dashed line indicates an odds ratio of 1.0.

These findings were consistently replicated in the independent testing set. The model maintained comparable discriminative performance, with an AUC of 0.733 (Figure 3A). Calibration curves again showed good concordance between predicted and observed risks, with low calibration error (Figure 3B). DCA confirmed sustained net clinical benefit across clinically meaningful thresholds (Figure 3C). Additionally, RCS analysis identified a significant nonlinear relationship between predicted risk and HAP occurrence (*P* for nonlinearity = 0.012), mirroring the trend observed in the training set (Figure 3D). Collectively, these results demonstrate that the model, primarily based on PIV and supplemented by PNI, achieved robust discrimination, reliable calibration, and consistent risk stratification across both derivation and validation cohorts.

### Performance comparison and selection of the optimal machine learning model

The predictive performance of six machine learning (ML) models for HAP was evaluated in the testing set (Table 2). Overall, all models demonstrated acceptable discrimination; however, clear differences were observed in their overall performance. Among the evaluated algorithms under the standardized default-parameter framework, LGBM showed the most favorable predictive performance, achieving an AUROC of 0.815, along with superior accuracy, recall, F1-score, and MCC, indicating balanced classification performance and high stability. Although the XGB model showed comparable discrimination, its recall and MCC values were slightly lower than those of the LGBM. The Random Forest and Decision Tree models demonstrated moderate performance, whereas the Support Vector Machine and Logistic Regression models yielded lower recall and F1-scores, reflecting a more conservative classification pattern associated with higher false-negative rates.

**Table 2:** Performance comparison of six machine learning models for HAP.

Model name	Accuracy	Prevalence	Recall	F1-score	MCC	AUROC	Presicion	Specificity	FNR	FPR
RFTEST	0.772	0.374	0.734	0.707	0.522	0.789	0.681	0.794	0.266	0.206
SVMTEST	0.743	0.374	0.563	0.621	0.434	0.730	0.692	0.850	0.438	0.150
DesicionTreeTEST	0.766	0.374	0.719	0.697	0.507	0.754	0.676	0.794	0.281	0.206
LGBMTEST	0.778	0.374	0.750	0.716	0.536	0.815	0.686	0.794	0.250	0.206
LogisticTEST	0.713	0.374	0.391	0.505	0.356	0.743	0.714	0.907	0.609	0.093
XGBTEST	0.772	0.374	0.703	0.698	0.515	0.816	0.692	0.813	0.297	0.187
Mean_scores	0.757	0.374	0.643	0.657	0.478	0.775	0.690	0.826	0.357	0.174

HAP, hospital-acquired pneumonia; RF, random forest; SVM, support vector machine; DT, decision tree; LGBM, light gradient boosting machine; XGB, extreme gradient boosting; Accuracy, overall classification accuracy; Prevalence, proportion of patients with hospital-acquired pneumonia; Recall, sensitivity (true positive rate); Precision, positive predictive value; Specificity, true negative rate; F1-score, harmonic mean of precision and recall; MCC, Matthews correlation coefficient; AUROC, area under the receiver operating characteristic curve; FNR, false negative rate; FPR, false positive rate.

**Figure 4:**
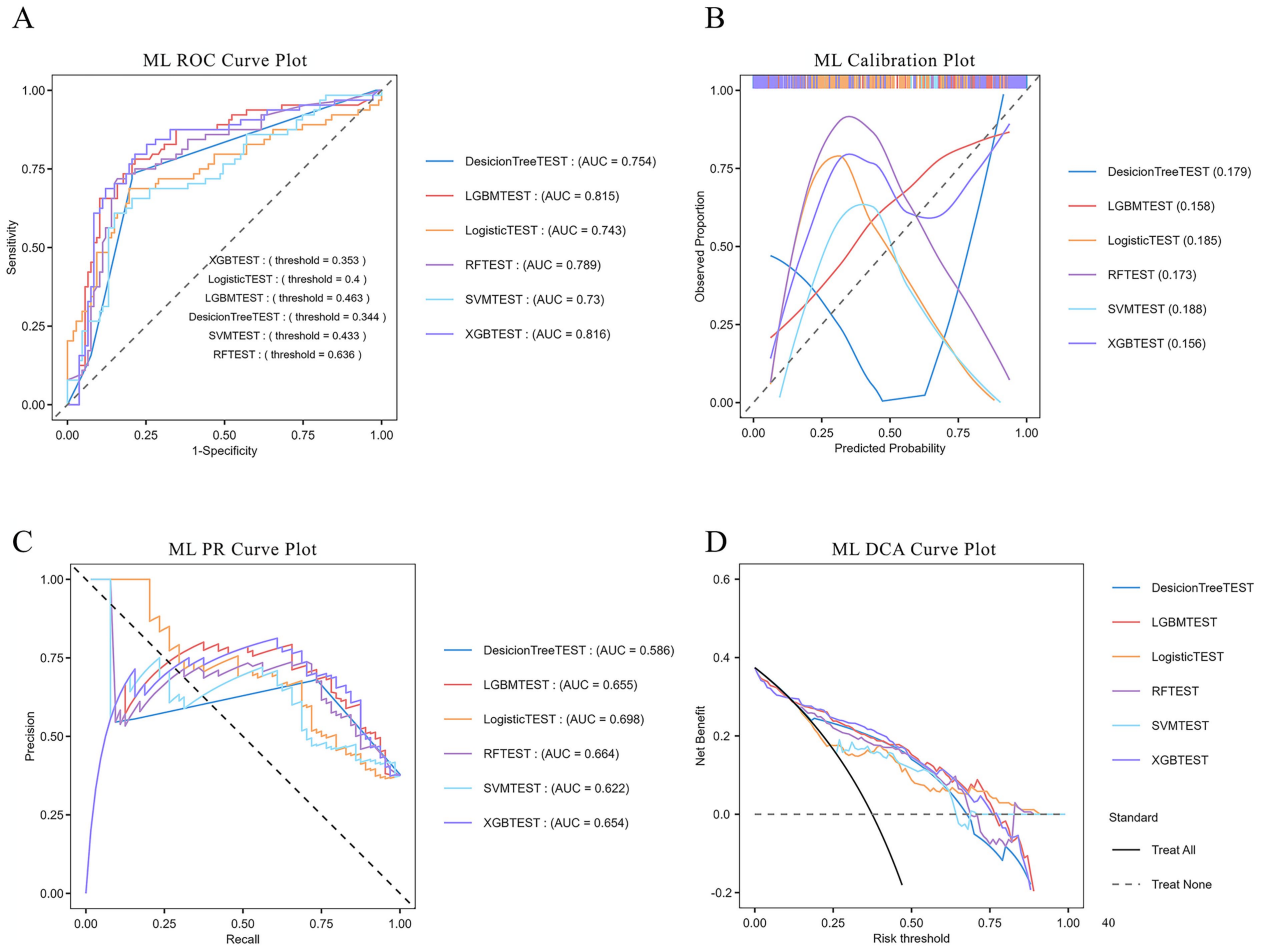
Comparative evaluation of ML models for predicting HAP. **(A)** Receiver operating characteristic (ROC) curves comparing the discriminative performance of six machine learning models in the testing cohort, including decision tree (DT), logistic regression (LR), random forest (RF), support vector machine (SVM), Light Gradient Boosting Machine (LGBM), and Extreme Gradient Boosting (XGB). The LGBM model achieved an area under the curve (AUC) of 0.815, which was among the highest across all models and comparable to XGB (AUC = 0.816), indicating superior discrimination for hospital-acquired pneumonia prediction. In contrast, DT (AUC = 0.754), LR (AUC = 0.743), and SVM (AUC = 0.730) demonstrated relatively lower discriminative performance. **(B)** Calibration curves illustrating the agreement between predicted probabilities and observed event rates for each model. The dashed diagonal line represents perfect calibration. Among the evaluated models, LGBM showed closer alignment with the ideal reference line across most predicted probability ranges and exhibited a relatively lower calibration error (0.158) compared with DT (0.179), LR (0.185), RF (0.173), and SVM (0.188), indicating more reliable probability estimation. XGB demonstrated comparable calibration performance with a calibration error of 0.156. **(C)** Precision–recall (PR) curves depicting the trade-off between precision and recall for each model in the testing cohort. The LGBM model achieved a precision–recall area under the curve (PR-AUC) of 0.655, reflecting a favorable balance between sensitivity and precision under class imbalance conditions. This performance was comparable to RF (PR-AUC = 0.664) and XGB (PR-AUC = 0.654), and superior to DT (0.586) and SVM (0.622). **(D)** Decision curve analysis (DCA) comparing the net clinical benefit of different machine learning models across a range of threshold probabilities. The LGBM model consistently yielded higher net benefit than the “treat-all” and “treat-none” strategies across most clinically relevant thresholds and showed comparable or superior net benefit relative to other models, supporting its potential utility for individualized risk-based clinical decision-making.

The comparative performance of these models is further illustrated in Figure 4. ROC curve analysis confirmed that the LGBM achieved one of the highest discriminative performances among all models (Figure 4A). Calibration analysis revealed closer concordance between predicted and observed HAP probabilities for the LGBM, with lower calibration error compared with other models (Figure 4B). Precision–recall curve analysis highlighted the robustness of the LGBM under class imbalance, demonstrating a favorable balance between sensitivity and precision (Figure 4C). Notably, decision curve analysis showed that the LGBM consistently provided greater net clinical benefit than the “treat-all” and “treat-none” strategies and performed comparably or better than other models across a wide range of clinically relevant threshold probabilities (Figure 4D).

### Cross-validation and robustness of the LGBM model

**Figure 5:**
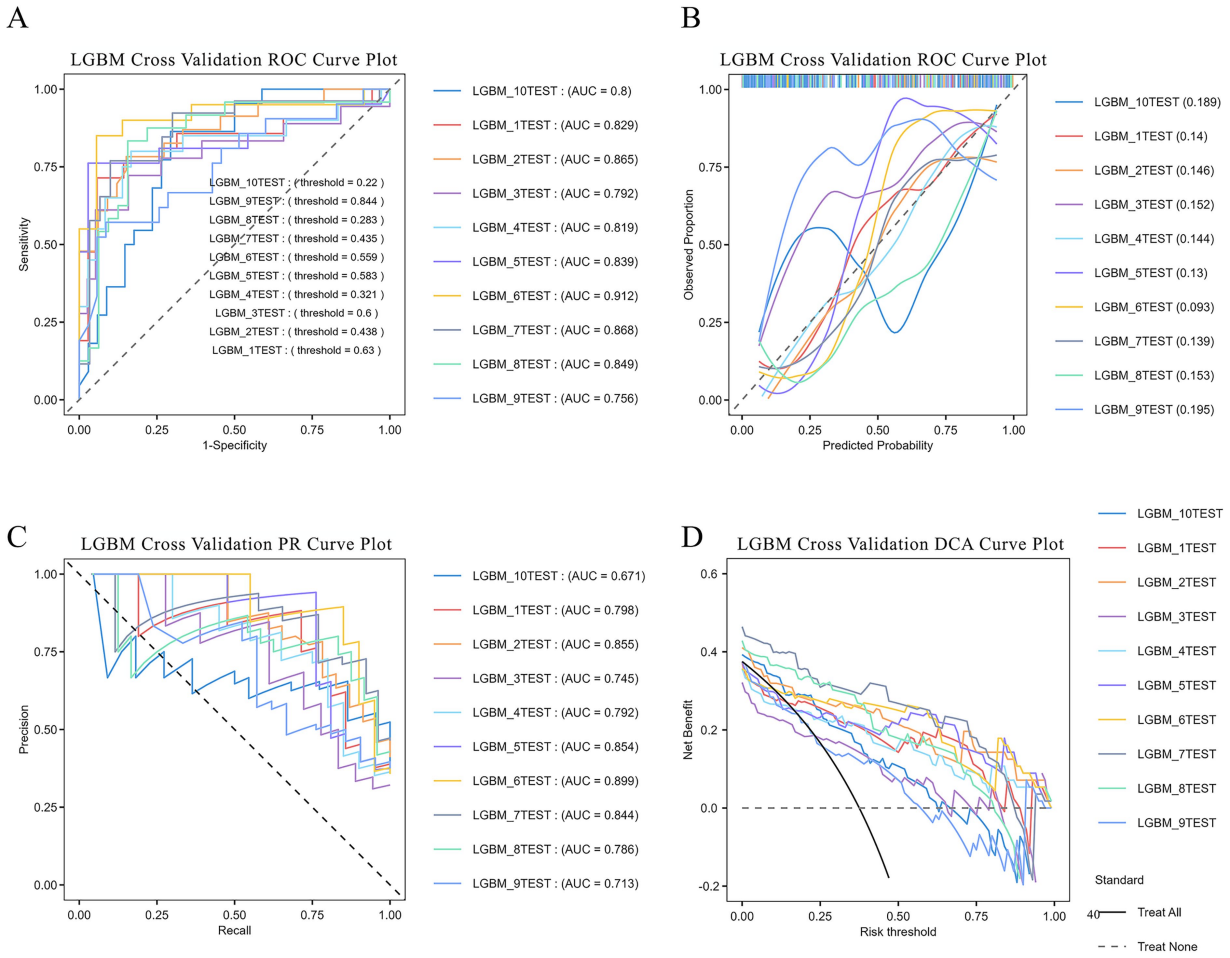
Internal validation of the LGBM model using 10-fold cross-validation. **(A)** Receiver operating characteristic (ROC) curves of the ten validation folds demonstrate consistently good discriminative ability of the LGBM model. The area under the curve (AUC) values across folds ranged from 0.756 to 0.912, with most folds achieving AUCs above 0.80, indicating stable and robust discrimination across different data partitions. **(B)** Calibration plots illustrate the agreement between predicted and observed HAP probabilities for each fold. Across the ten folds, calibration curves generally followed the ideal diagonal reference line, with calibration errors ranging approximately from 0.093 to 0.195, suggesting acceptable probability estimation and limited systematic over- or underestimation of risk. **(C)** Precision–recall (PR) curves show the model’s performance under outcome imbalance. The PR-AUC values varied across folds, ranging from 0.671 to 0.899, indicating that the LGBM model maintained a favorable balance between precision and recall in most validation subsets. **(D)** Decision curve analysis (DCA) demonstrates that, across all ten folds, the LGBM model provided a positive net clinical benefit over the “treat-all” and “treat-none” strategies across a broad range of clinically relevant threshold probabilities, supporting its potential utility for individualized risk-based decision-making.

To further assess the robustness and stability of the LGBM model, 10-fold cross-validation was conducted using the training cohort, with results summarized in Table 3 and illustrated in Figure 5. Across the ten folds, the model demonstrated consistently strong predictive performance, with accuracy ranging from 0.696 to 0.893 and AUROC values between 0.756 and 0.913 (Figure 5A). Most folds achieved AUROC values above 0.80, with a mean AUROC of 0.833, indicating stable and reproducible discrimination across distinct data partitions.

**Table 3:** Performance of the LGBM model evaluated by 10-fold cross-validation.

Model name	Accuracy	Prevalence	Recall	F1-score	MCC	AUROC	Presicion	Specificity	FNR	FPR
LGBM_1TEST	0.768	0.375	0.762	0.711	0.522	0.829	0.667	0.771	0.238	0.229
LGBM_2TEST	0.804	0.411	0.739	0.756	0.592	0.865	0.773	0.848	0.261	0.152
LGBM_3TEST	0.750	0.321	0.722	0.650	0.464	0.792	0.591	0.763	0.278	0.237
LGBM_4TEST	0.804	0.357	0.650	0.703	0.562	0.819	0.765	0.889	0.350	0.111
LGBM_5TEST	0.839	0.375	0.762	0.780	0.654	0.839	0.800	0.886	0.238	0.114
LGBM_6TEST	0.893	0.357	0.850	0.850	0.767	0.913	0.850	0.917	0.150	0.083
LGBM_7TEST	0.804	0.464	0.692	0.766	0.610	0.868	0.857	0.900	0.308	0.100
LGBM_8TEST	0.750	0.429	0.583	0.667	0.486	0.849	0.778	0.875	0.417	0.125
LGBM_9TEST	0.696	0.375	0.667	0.622	0.373	0.756	0.583	0.714	0.333	0.286
LGBM_10TEST	0.714	0.393	0.500	0.579	0.382	0.800	0.688	0.853	0.500	0.147
Mean_Scores	0.782	0.386	0.693	0.708	0.541	0.833	0.735	0.842	0.307	0.158

Fold, cross-validation fold; Accuracy, overall classification accuracy; Recall, sensitivity (true positive rate); Precision, positive predictive value; Specificity, true negative rate; F1-score, harmonic mean of precision and recall; MCC, Matthews correlation coefficient; AUROC, area under the receiver operating characteristic curve; FPR, false positive rate; FNR, false negative rate.

Beyond discrimination, the LGBM model exhibited favorable classification balance, calibration, and potential clinical relevance. The mean recall and F1-score were 0.693 and 0.708, respectively, while the mean MCC was 0.541, demonstrating consistent agreement between predicted and observed outcomes (Table 3). Calibration analysis revealed good concordance between predicted and observed HAP probabilities, with calibration errors ranging from 0.093 to 0.195 across folds (Figure 5B). Precision–recall curve analysis further confirmed stable model performance, with PR-AUC values ranging from 0.671 to 0.899 (Figure 5C). DCA showed that the model consistently produced positive net clinical benefit compared with the “treat-all” and “treat-none” strategies across all clinically relevant threshold probabilities (Figure 5D).

### Model interpretability based on SHAP analysis

**Figure 6:**
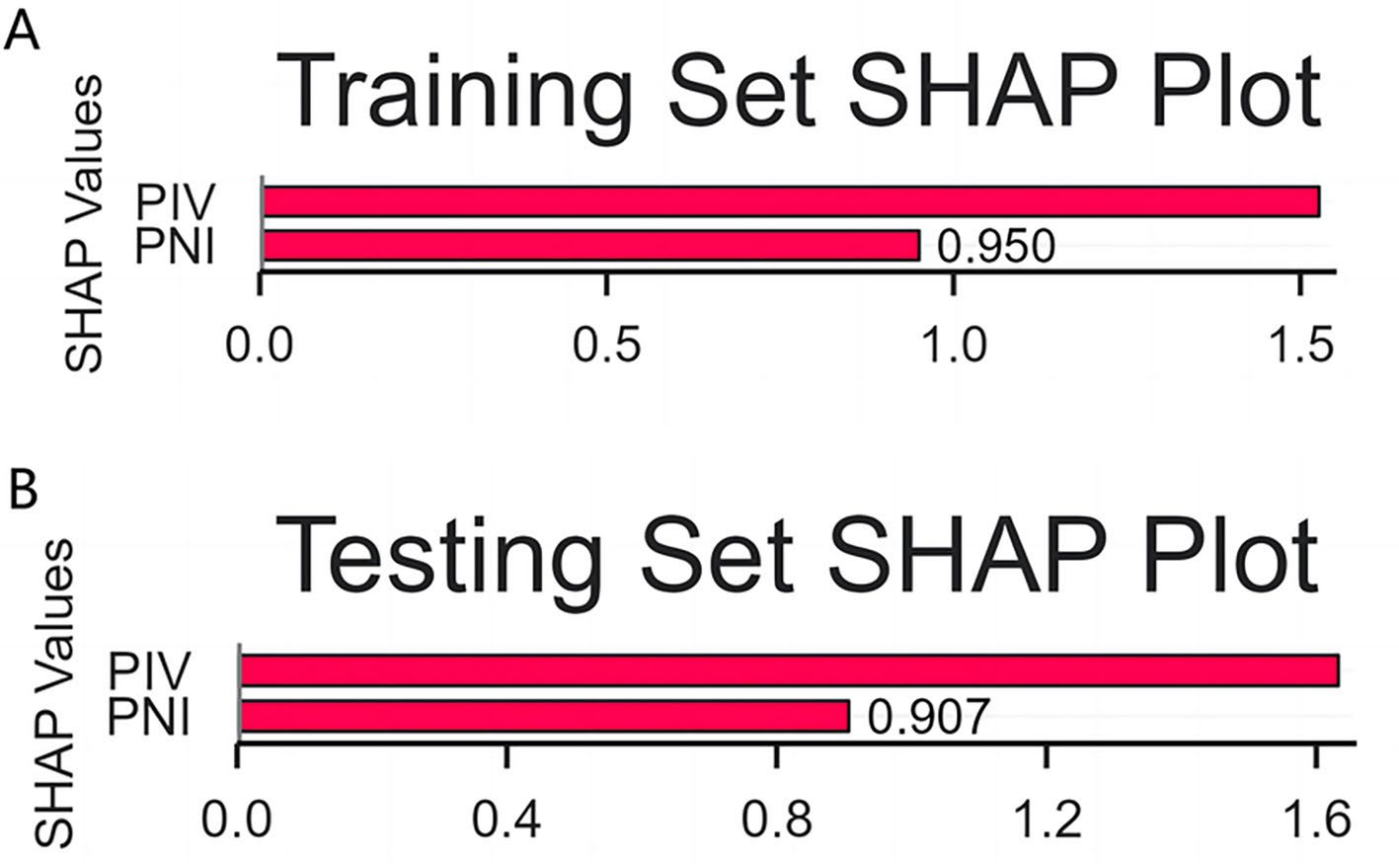
SHAP analysis of feature importance in the training and testing sets. This figure presents the Shapley additive explanations (SHAP) analysis illustrating the relative importance of predictors in the machine learning–based model for hospital-acquired pneumonia (HAP) risk prediction. **(A)** In the training set, the Pan-Immune-Inflammation Value (PIV) demonstrated the highest mean absolute SHAP value (approximately 1.55), indicating that it was the most influential variable driving model predictions. The Prognostic Nutritional Index (PNI) also showed a substantial contribution, with a mean SHAP value of 0.950, ranking as the second most important feature. **(B)** In the testing set, a highly consistent pattern of feature importance was observed. PIV remained the dominant predictor, with a mean absolute SHAP value of approximately 1.60, while PNI continued to exert a strong but comparatively smaller effect, with a mean SHAP value of 0.907. The concordant ranking and similar magnitude of SHAP values across the training and testing sets indicate stable feature importance and support the robustness of the model’s decision-making process. Overall, these findings highlight the central role of inflammatory burden, complemented by nutritional status, in predicting the risk of HAP after traumatic brain injury.

To enhance model interpretability, SHAP analysis was employed to quantify the contribution of individual predictors to HAP risk. As shown in Figure 6, PIV emerged as the most influential feature in both the training and testing cohorts. In the training cohort, PIV exhibited a mean absolute SHAP value of approximately 1.55, markedly higher than that of PNI, which had a mean SHAP value of 0.950, indicating a dominant contribution of inflammatory burden to model predictions.

This feature importance pattern was consistently replicated in the testing cohort. PIV remained the strongest predictor, with a mean absolute SHAP value of approximately 1.60, while PNI continued to provide a meaningful complementary contribution, with a mean SHAP value of 0.907. The close consistency in both ranking and magnitude of SHAP values across cohorts demonstrates the stability and robustness of the model. Collectively, these findings indicate that systemic immune–inflammatory status serves as the primary driver of prediction, whereas nutritional reserve exerts a secondary yet important effect, thereby supporting the biological plausibility and clinical relevance of this integrated inflammatory–nutritional prediction framework.

### Risk stratification of functional prognosis based on model-predicted probability

The sensitivity analyses were performed in the training set to evaluate whether the association between the model-derived risk estimate and HAP remained robust after adjustment for available baseline covariates (Supplementary Table S1). The continuous LGBM model score remained significantly associated with HAP in the unadjusted model, the partially adjusted model, and the fully adjusted model, with the association persisting in Model 3 (OR = 10.237, 95% CI 1.035–101.223, *p* = 0.047). When the model-predicted values were categorized into four groups, higher-risk categories showed progressively increased odds of HAP, and the trend remained significant after full adjustment (*P* for trend = 0.005). These findings support the robustness of the model-derived predictive association after accounting for measured baseline covariates.

**Figure 7:**
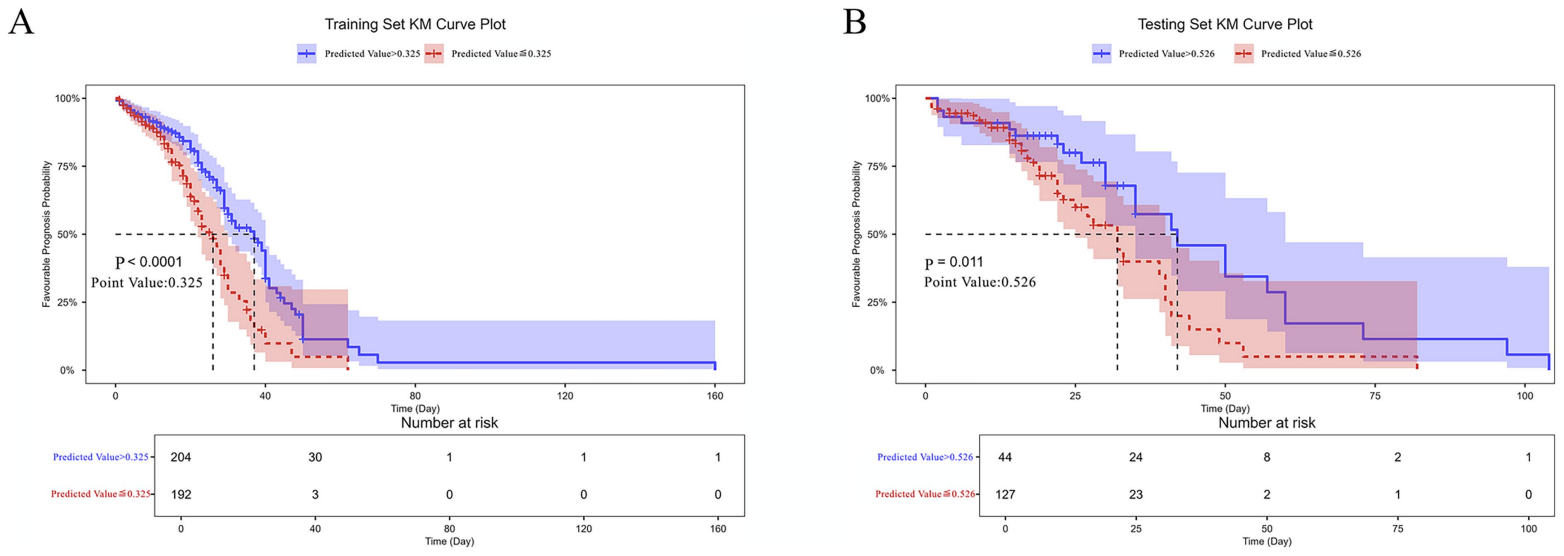
Kaplan–Meier curves of favorable functional prognosis stratified by model-predicted risk. This figure shows Kaplan–Meier (KM) curves illustrating the association between prediction model–derived risk stratification and functional outcomes at discharge, assessed using the modified Rankin Scale (mRS). A favorable prognosis was defined as an mRS score of 0–2, whereas an unfavorable prognosis was defined as an mRS score of 3–6. Patients were dichotomized into high-risk and low-risk groups according to the optimal cutoff value of the model-predicted probability. The y-axis represents the cumulative probability of achieving a favorable functional outcome, and the x-axis represents time from onset (days). Shaded areas indicate 95% confidence intervals, and the numbers at risk at predefined time points are displayed below each plot. **(A)** In the training set, the KM curves demonstrated a clear and early separation between the high-risk (model predicted value 0.325) and low-risk (model predicted value≤0.325) groups. Patients classified as high risk exhibited a significantly lower probability of favorable functional prognosis compared with those in the low-risk group throughout hospitalization (log-rank test, *p* < 0.0001). At baseline, 204 patients were included in the high-risk group and 192 in the low-risk group, with a more rapid decline in the number at risk observed in the high-risk group over time. **(B)** In the testing set, the prognostic stratification effect of the model was consistently reproduced. Patients in the high-risk (model predicted value 0.526) group showed a persistently reduced probability of favorable functional outcome compared with low-risk (model predicted value≤0.526) patients, and the difference between the two curves remained statistically significant (log-rank test, *p* = 0.011). At baseline, 44 patients were classified as high risk and 127 as low risk. Despite the smaller sample size, the model retained its ability to discriminate functional prognosis across follow-up.

As an exploratory in-hospital prognostic analysis, K-M curves were used to visualize separation in favorable discharge outcome across model-defined risk groups (Figure 7). In the training set, patients were divided into high-risk and low-risk groups using a predicted probability cutoff of 0.325. This cutoff was selected for prognostic stratification in the KM analysis and therefore differs from the ROC-based cutoff used for HAP classification. The KM curves demonstrated a clear separation between the two groups, with high-risk patients exhibiting a significantly lower cumulative probability of achieving a favorable functional outcome (mRS 0–2) compared with low-risk patients (log-rank *p* < 0.0001; Figure 7A). This divergence emerged early after admission and persisted throughout hospitalization.

Consistent results were observed in the independent testing set. Using an optimal cutoff value of 0.526, high-risk patients again showed a significantly lower probability of favorable functional outcome compared with those classified as low risk (log-rank *p* = 0.011; Figure 7B). This value was likewise derived for prognostic stratification rather than for ROC-based diagnostic classification. Despite the smaller sample size, the model maintained its ability to stratify functional prognosis across the hospitalization period, confirming the robustness of its prognostic performance.

## Discussion

In this two-centers retrospective study, we developed and validated an inflammatory–nutritional ML model for predicting HAP after TBI and for stratifying patients by functional prognosis. Several main findings emerged. First, the model integrating PIV and PNI showed favorable predictive performance, with an AUROC of 0.815 in the independent testing cohort, together with good calibration and favorable clinical net benefit. Second, within the present exploratory comparison using standardized default settings, LGBM showed the best overall performance, and its stability was further supported by 10-fold cross-validation, with a mean AUROC of approximately 0.83. Third, SHAP analysis identified PIV as the dominant contributor to model predictions, whereas PNI provided a stable complementary contribution, supporting the biological plausibility of the inflammatory–nutritional framework. Finally, model-derived risk stratification was associated not only with HAP occurrence but also with functional outcomes at discharge, as high-risk patients showed a significantly lower probability of favorable prognosis (mRS 0–2) in both the training and testing cohorts. Collectively, these findings suggest that combining systemic inflammatory burden and nutritional–immune status within an interpretable ML framework may provide a clinically meaningful tool for early risk assessment and prognostic stratification after TBI.

The biological relevance of this model is supported by the established interplay between systemic inflammation, nutritional–immune status, and post-TBI complications (15–17). TBI induces a complex biphasic immune response characterized by early hyperinflammation followed by prolonged immune suppression, often referred to as TBI-induced immunodepression. This process involves excessive release of pro-inflammatory cytokines, including tumor necrosis factor-*α*, interleukin-6, and interleukin-1β, together with lymphocyte apoptosis and impaired antigen presentation, thereby increasing susceptibility to secondary infections such as HAP (18, 19). The PIV, which integrates neutrophil, platelet, monocyte, and lymphocyte counts, captures multiple dimensions of this dysregulated immune response (20). Elevated neutrophil and monocyte counts reflect exaggerated innate immune activation, whereas relative lymphopenia indicates compromised adaptive immunity and diminished immune surveillance (21–23). Platelets may further contribute through immunothrombotic and inflammatory pathways, promoting endothelial injury and microvascular dysfunction that could increase the risk of HAP (24). Nutritional–immune status represents another relevant dimension (6). The PNI, derived from serum albumin and lymphocyte count, reflects both protein reserves and immune competence (10). Hypoalbuminemia is associated with systemic inflammation, oxidative stress, and increased capillary permeability, while lymphopenia indicates impaired cellular immunity (12, 25). Together, these abnormalities may indicate a state of nutritional–immune exhaustion that amplifies infection risk and hinders neurological recovery. By integrating PIV and PNI, the present model captures two biologically complementary pathways underlying both HAP susceptibility and poor functional prognosis after TBI.

Previous studies of HAP after TBI have mainly focused on conventional clinical and injury-related variables. For example, Matthew et al. identified age and GCS as key predictors of pneumonia risk (2); whereas David et al. emphasized injury severity, mechanical ventilation, and level of consciousness, most often examined using conventional regression-based models (26). Although these studies have advanced understanding of post-TBI pneumonia risk, many models have shown only modest predictive performance and lacked independent center-based validation, limiting their generalizability and clinical applicability (27, 28). In addition, most prior tools provide limited interpretability and do not fully capture the biological mechanisms underlying individual risk estimates. Inflammatory and nutritional determinants, which are closely linked to post-TBI immune dysregulation and infection susceptibility, have often been underrepresented or evaluated only through isolated biomarkers. Studies of single markers such as leukocyte count, neutrophil-to-lymphocyte ratio, or serum albumin have reported associations with pneumonia risk (29–31); however, such variables alone are unlikely to reflect the full interaction between systemic inflammation and nutritional–immune reserve after TBI. In contrast, the present study integrates the composite indices PIV and PNI within an ML framework that can model nonlinear relationships. The two-center design with independent center-based validation further strengthens the robustness of the findings. In addition, SHAP analysis improves transparency by providing biologically meaningful explanations for individual predictions. A more direct comparison with recently published TBI-HAP prediction studies also helps position the incremental value of the present model. Li et al. reported AUCs of 0.818 and 0.819 using a single-center model combining clinical and CT imaging variables, with logistic regression identified as the optimal algorithm (27). By comparison, our model achieved a similar AUROC of 0.815 in an independent testing cohort, despite relying only on two composite inflammatory–nutritional indices derived from routinely available admission laboratory data. In another recent study, Wei et al. developed a nomogram based on six conventional predictors and reported an AUC of 0.790. Relative to that model, our LGBM model showed numerically higher discrimination, while also providing SHAP-based interpretability and functional prognostic stratification (28). These comparisons should be interpreted as indirect literature-based benchmarking rather than evidence of definitive superiority, because no head-to-head validation or formal statistical comparison with prior models was feasible.

Comparison with logistic regression further supports the practical value of the ML-based approach. Although LR achieved acceptable discrimination, its substantially lower recall and higher false-negative rate may limit its usefulness for early HAP screening after TBI. By contrast, LGBM showed a more balanced performance profile and therefore greater potential utility in a screening-oriented setting. Importantly, this added complexity should be understood mainly at the algorithmic level rather than as additional clinical burden, because both models were built from the same two routinely available predictors, PIV and PNI. Thus, the main trade-off in the present study is not between simple and complex data collection, but between the greater transparency of LR and the improved screening performance of LGBM, with SHAP partially mitigating the latter model’s reduced intrinsic interpretability (Supplementary Table S2).

The findings of this study also have several potential clinical implications. First, the proposed inflammatory–nutritional ML model enables early individualized risk assessment for HAP using routine laboratory data obtained within 24 h of admission. This may help clinicians identify high-risk patients early and initiate preventive strategies, such as intensified respiratory care, earlier mobilization, optimized nutritional support, and closer infection surveillance. Second, integration of PIV and PNI provides a biologically interpretable framework linking immune–inflammatory dysregulation and nutritional reserve with infection susceptibility and functional recovery, which may support multidisciplinary decision-making in neurocritical care. Third, the ability of the model to stratify functional prognosis extends its value beyond infection prediction alone and may assist in risk communication, resource allocation, and individualized rehabilitation planning. Owing to its reliance on easily obtainable variables, the model could potentially be incorporated into clinical workflows or electronic health record–based decision support systems. Clinically, the model may serve as an admission-based HAP prediction tool using routinely available laboratory data obtained within 24 h of admission. The predicted risk probability may help identify patients who warrant closer surveillance and more proactive preventive management. In our study, model-based cutoffs of 0.325 in the training set and 0.526 in the testing set provided initial reference points for risk stratification; however, the optimal operational threshold may vary across settings and should be locally calibrated before routine implementation. To further improve clinical usability, we provided a proposed implementation workflow and threshold-guided action pathway in the Supplementary materials. In addition, a static nomogram and a web-based dynamic nomogram were developed as implementation aids for individualized risk visualization (https://caichenzhu.shinyapps.io/dynnomapp/). These tools are intended to facilitate exploratory clinical illustration.

### Limitations and future directions

Several limitations should be acknowledged. First, the retrospective design and complete-case analysis may have introduced selection bias, reduced statistical power, and limited causal inference; future studies should prospectively validate the model and compare complete-case and multiple-imputation strategies when appropriate. Second, although the model was evaluated in an independent cohort from a second hospital, both centers were located within the same province and had overlapping enrollment periods. Therefore, the present study should be interpreted as providing independent center-based validation rather than geographically broad external or true temporal validation, and broader validation in geographically and clinically diverse populations, together with model recalibration in substantially different healthcare settings, remains necessary. Third, no formal sensitivity analyses were performed for additional clinical covariates, so residual confounding cannot be excluded. Accordingly, the present findings should be interpreted as reflecting predictive associations within the current admission-based framework rather than causal effects. In addition, because HAP ascertainment relied partly on retrospective clinical documentation and radiological reports, some degree of outcome misclassification may have occurred despite the use of predefined diagnostic criteria across both centers. In addition, because outcome assessment was retrospective and no formal inter-rater reliability analysis was available, some clinically unrecognized or undocumented HAP cases may have been missed. Fourth, the model was intentionally based on laboratory data obtained within 24 h of admission to support early risk stratification before HAP onset, but this design cannot capture dynamic inflammatory–nutritional changes over time. Future studies should compare baseline-based and longitudinal models, and larger multicenter datasets with richer longitudinal or multimodal data may also permit evaluation of deep learning approaches, provided that interpretability and clinical simplicity are preserved. Finally, although SHAP improved model interpretability, the clinical impact of model-guided interventions was not assessed and should be examined in prospective implementation studies.

## Conclusion

This multicenter study demonstrated that an interpretable inflammatory–nutritional ML model integrating PIV and PNI supported accurate prediction of HAP after TBI within the present two-center study context. Integrating immune–inflammatory burden with nutritional–immune reserve not only enhanced risk discrimination and clinical net benefit but also effectively stratified patients by functional prognosis. The inclusion of SHAP analysis further improved model transparency and biological plausibility, supporting its potential value for early risk assessment and future individualized decision-support in patients with TBI, pending prospective validation.

## Data Availability

The raw data supporting the conclusions of this article will be made available by the authors, without undue reservation.
