# Supplementary material for: An inflammatory–nutritional machine learning model for risk stratification of hospital-acquired pneumonia in traumatic brain injury: a multicenter study
Source: Front Nutr. 2026 May 28;13:1785139. doi: 10.3389/fnut.2026.1785139 (PMC13253410; doi:10.3389/fnut.2026.1785139)
Supplement: Supplementary file 1 [file Data_Sheet_1.zip › Supplementary Table 2.docx]

**Supplemental Table S2 Structured Comparison between Logistic Regression and LGBM in the Testing Set**

| **Domain** | **Logistic Regression** | **LGBM** | **Clinical interpretation** |
| --- | --- | --- | --- |
| **Input variables** | PIV + PNI | PIV + PNI | Same data burden |
| **AUROC** | 0.743 | 0.815 | LGBM showed better discrimination |
| **Recall** | 0.391 | 0.75 | LGBM identified substantially more high-risk patients |
| **Specificity** | 0.907 | 0.794 | LR was more conservative, with fewer false positives |
| **Precision** | 0.714 | 0.686 | Similar positive predictive value |
| **F1-score** | 0.505 | 0.716 | LGBM showed better overall balance |
| **MCC** | 0.356 | 0.536 | LGBM showed stronger overall agreement |
| **False-negative rate** | 0.609 | 0.25 | LR missed substantially more HAP cases |
| **Calibration error** | 0.185 | 0.158 | LGBM provided slightly more reliable probability estimates |
| **DCA** | Lower/comparable | Greater/comparable | LGBM showed better decision support potential |
| **Interpretability** | High | Moderate, improved by SHAP | LR is more transparent; LGBM is partly interpretable with SHAP |
| **Practical use** | Conservative rule-in model | Screening-oriented early warning model | Choice depends on clinical priority |

**LGBM: Light Gradient Boosting Machine, LR:Logistic Regression, PIV: Pan Immune Inflammation Value, PNI:Prognostic Nutritional Index, AUROC: Area Under the Receiver Operating Characteristic Curve, MCC: Matthews Correlation Coefficient, DCA: Decision Curve Analysis, HAP: Hospital-Acquired Pneumonia**
