# Supplementary material for: An inflammatory–nutritional machine learning model for risk stratification of hospital-acquired pneumonia in traumatic brain injury: a multicenter study
Source: Front Nutr. 2026 May 28;13:1785139. doi: 10.3389/fnut.2026.1785139 (PMC13253410; doi:10.3389/fnut.2026.1785139)
Supplement: Supplementary file 1 [file Data_Sheet_1.zip › Supplementary File 1.docx]

**Supplementary Materials**

**An Inflammatory–Nutritional Machine Learning Model for Risk Stratification of Hospital-Acquired Pneumonia in Traumatic Brain Injury: A Multicenter Study**

This supplementary document provides an illustrative workflow for clinical implementation of the admission-based LGBM model, a threshold-guided action table, and nomogram-based tools for early HAP risk stratification after traumatic brain injury.

**Supplementary Figure S2. Proposed workflow for admission-based clinical implementation of the LGBM model for early HAP risk stratification after traumatic brain injury**


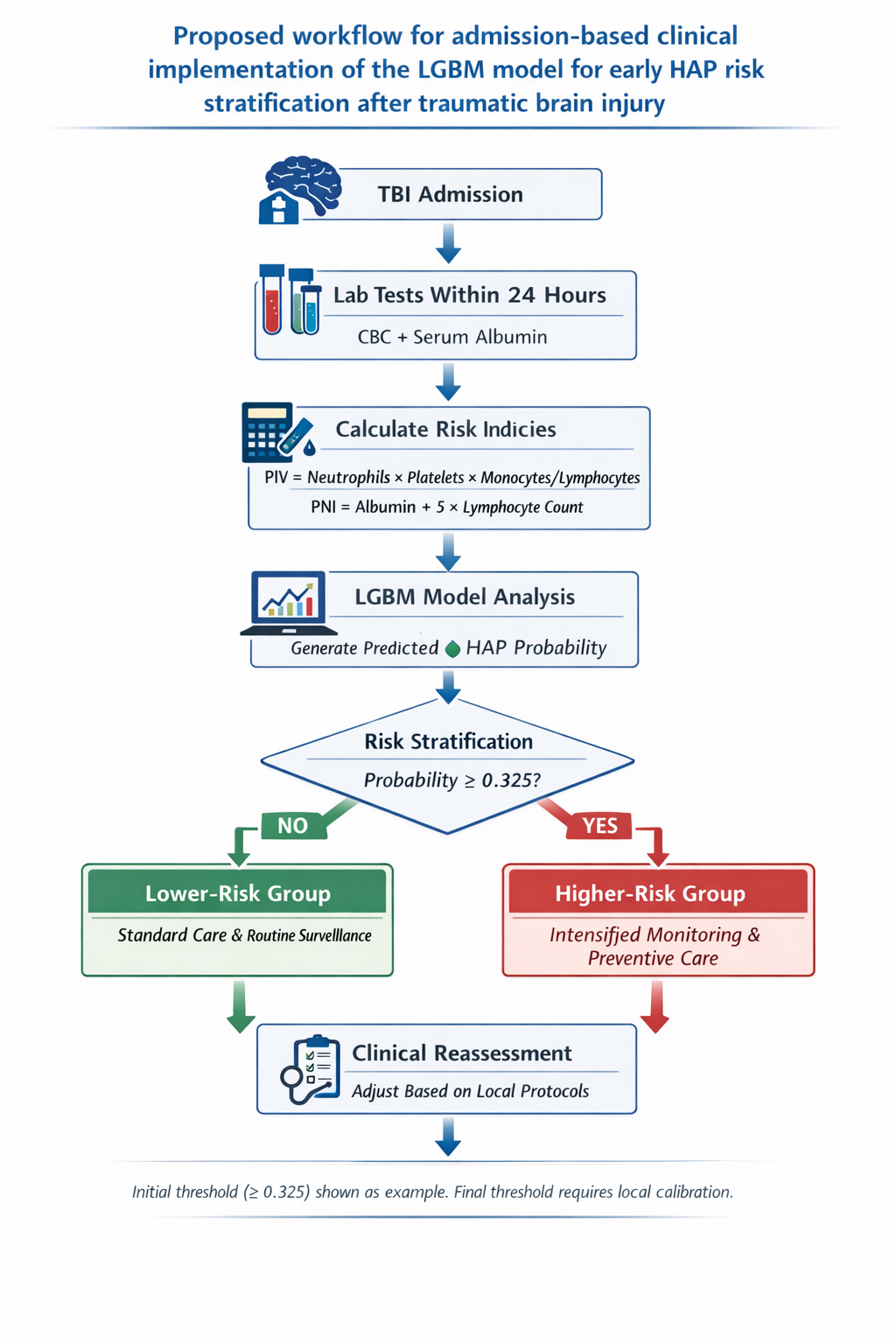


**Figure legend:** Within 24 hours of admission, routinely available laboratory data are used to calculate the PIV and PNI, which are then entered into the LGBM model to generate an individualized probability of HAP. The derivation-cohort threshold is presented as an initial operational reference to support early risk stratification. Patients above this threshold may be considered for closer surveillance and more proactive preventive management, whereas patients below this threshold may continue with standard care. This workflow is illustrative rather than prospectively validated, and final operational thresholds should be locally recalibrated according to clinical priorities and resource availability.

**Supplementary Table S3 Illustrative threshold-guided clinical actions for model-assisted early risk stratification**

| **Model-predicted probability of HAP** | **Suggested interpretation** | **Illustrative clinical action** | **Rationale** |
| --- | --- | --- | --- |
| < 0.325 | Lower-risk group | Continue standard care and routine surveillance according to institutional practice | Lower predicted risk; no additional model-triggered action required |
| ≥ 0.325 | Higher-risk group | Consider closer respiratory surveillance, intensified airway care, earlier mobilization, nutritional optimization, and more proactive preventive management | Higher predicted risk; consider earlier preventive attention before overt HAP develops |

**HAP: hospital-acquired pneumonia, LGBM: Light Gradient Boosting Machine, PIV: Pan Immune Inflammation Value, PNI: Prognostic Nutritional Index.**

**Table Legend:** The threshold shown in this table is based on the derivation cohort and is presented as an initial operational reference for illustrative purposes. It should not be interpreted as a universal cutoff. The final threshold for clinical implementation may vary according to local calibration, resource availability, and the preferred balance between sensitivity and false-positive alerts. The suggested actions are conceptual decision-support pathways rather than prospectively validated intervention recommendations.

**Supplementary Figure S3 Illustrative nomogram for individualized risk estimation based on admission inflammatory–nutritional variables**


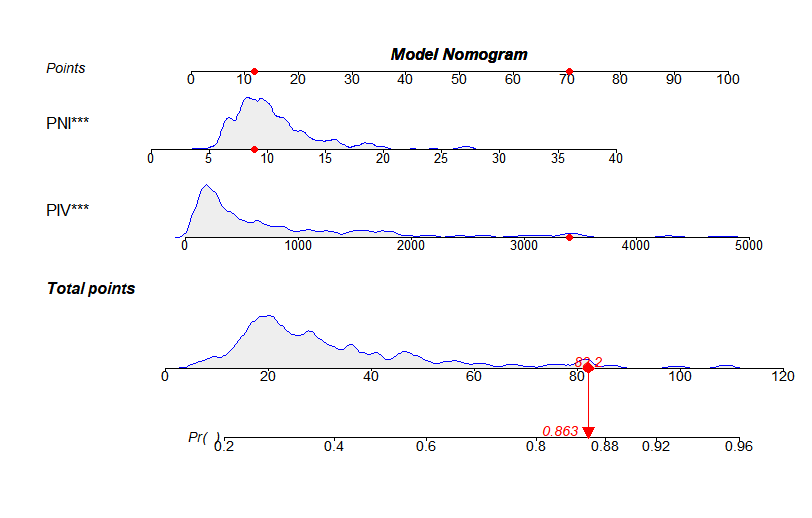


**Figure legend:** This nomogram is presented as an adjunctive implementation aid to facilitate bedside visualization of individualized HAP risk estimation based on the same admission inflammatory–nutritional framework. The red markings illustrate an example from a randomly selected patient in the training cohort. In this case, a PNI of approximately 9 corresponds to about 12 points, and a PIV of approximately 3,400 corresponds to about 70 points, yielding a total score of 82 points. This total score maps to an estimated predicted probability of HAP of 0.863. The figure is intended to improve operational interpretability of the model output, but it does not replace local calibration or prospective validation before routine clinical implementation.

## Supplementary Note S1 Web-based Dynamic Nomogram for Operational Illustration

A web-based dynamic nomogram was additionally developed to support interactive clinical illustration and exploratory use. The tool allows users to enter individual PIV and PNI values and obtain an estimated probability of HAP in real time. It is available at: [**https://caichenzhu.shinyapps.io/dynnomapp/**](https://caichenzhu.shinyapps.io/dynnomapp/)**.** This web-based tool is presented as an implementation aid to complement the main LGBM workflow. The displayed probability should be interpreted together with local clinical judgment, and any operational threshold used in practice should be prospectively validated and locally calibrated before routine deployment.
