# Supplementary material for: An inflammatory–nutritional machine learning model for risk stratification of hospital-acquired pneumonia in traumatic brain injury: a multicenter study
Source: Front Nutr. 2026 May 28;13:1785139. doi: 10.3389/fnut.2026.1785139 (PMC13253410; doi:10.3389/fnut.2026.1785139)
Supplement: Supplementary file 1 [file Data_Sheet_1.zip › Supplementary File 2.docx]

**Supplementary Materials**

**Comparison of the inflammatory–nutritional model with a clinical model and a combined model**

**Supplementary Figure ROC comparison of the inflammatory–nutritional model 1 (PIV + PNI), a simple clinical model 2 (Age + GCS), and a combined model 3 (PIV + PNI + Age + GCS).**


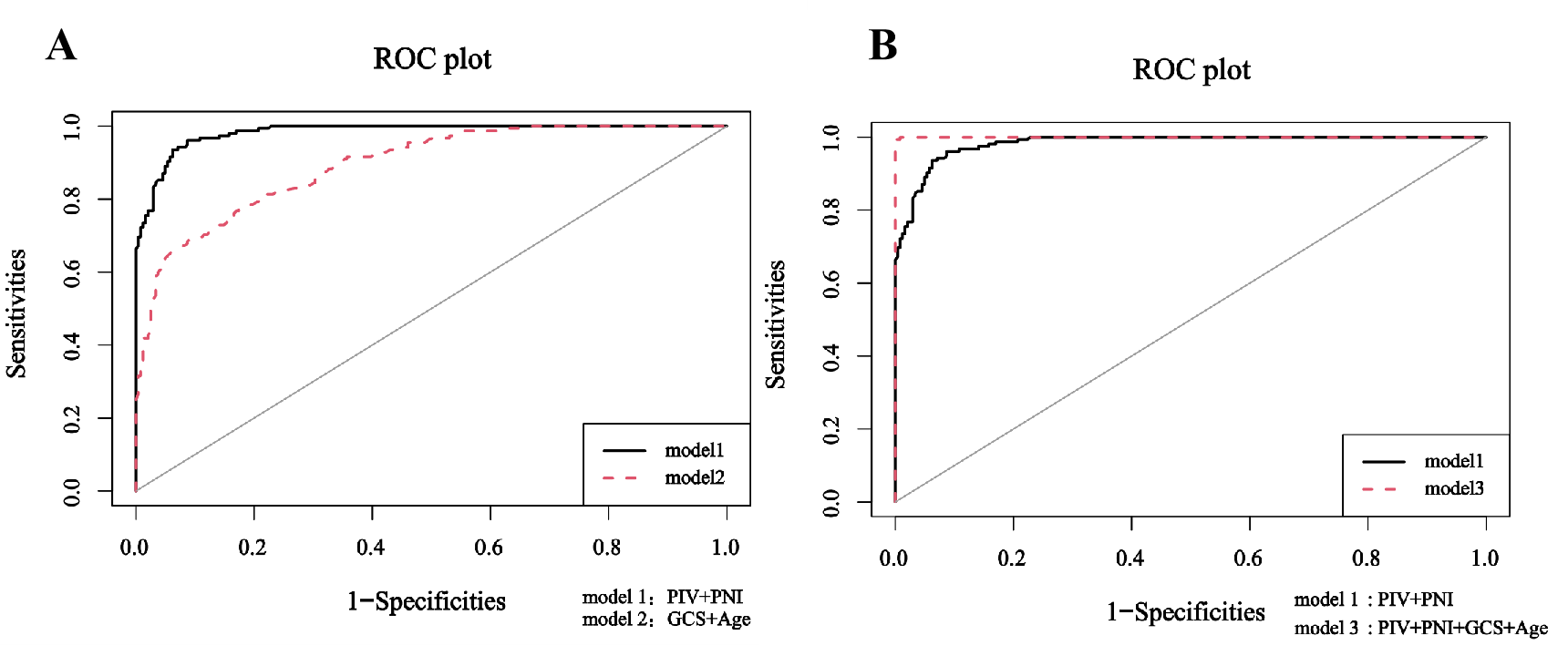


**Figure legend:** (A) ROC curves comparing the inflammatory–nutritional model (model 1: PIV + PNI) with the simple clinical model (model 2: Age + GCS). The inflammatory–nutritional model showed clearly better discrimination than the clinical model. Consistently, reclassification analyses demonstrated significantly negative NRI and IDI values for the clinical model relative to the inflammatory–nutritional model, indicating inferior predictive performance. (B) ROC curves comparing the inflammatory–nutritional model (model 1: PIV + PNI) with the combined model (model 3: PIV + PNI + Age + GCS). The combined model showed slightly improved discrimination. This was supported by significantly positive NRI and IDI values, suggesting that Age and GCS provide additional complementary predictive information when added to the inflammatory–nutritional framework. PIV, Pan-Immune-Inflammation Value; PNI, Prognostic Nutritional Index; GCS, Glasgow Coma Scale; ROC, receiver operating characteristic; NRI, net reclassification improvement; IDI, integrated discrimination improvement.

**Supplementary Table NRI and IDI analyses comparing the inflammatory–nutritional model with the clinical model and the combined model**

| **Comparison** | **Metric** | **Value** | **SE** | **Z value** | **95% CI** | **P value** |
| --- | --- | --- | --- | --- | --- | --- |
| Model 1 vs Model 2 (PIV + PNI vs Age + GCS) | NRI | -0.6799 | 0.0971 | -7.0007 | -0.8703 to -0.4896 | <0.001 |
|  | IDI | -0.2247 | 0.0282 | -7.9585 | -0.2800 to -0.1693 | <0.001 |
| Model 1 vs Model 3 (PIV + PNI vs PIV + PNI + Age + GCS) | NRI | 1.2441 | 0.0805 | 15.4447 | 1.0862 to 1.4019 | <0.001 |
|  | IDI | 0.1973 | 0.0144 | 13.7331 | 0.1692 to 0.2255 | <0.001 |

**Abbreviations:** PIV, Pan-Immune-Inflammation Value; PNI, Prognostic Nutritional Index; GCS, Glasgow Coma Scale; NRI, net reclassification improvement; IDI, integrated discrimination improvement; SE, standard error; CI, confidence interval.

**Interpretation:** Negative NRI and IDI values in the comparison between Model 1 and Model 2 indicate that the simple clinical model based on Age and GCS performed worse than the inflammatory–nutritional model. Positive NRI and IDI values in the comparison between Model 1 and Model 3 indicate that adding Age and GCS to PIV and PNI further improved predictive performance.
