# Supplementary material for: An inflammatory–nutritional machine learning model for risk stratification of hospital-acquired pneumonia in traumatic brain injury: a multicenter study
Source: Front Nutr. 2026 May 28;13:1785139. doi: 10.3389/fnut.2026.1785139 (PMC13253410; doi:10.3389/fnut.2026.1785139)
Supplement: Supplementary file 1 [file Data_Sheet_1.zip › Supplementary Table 1.docx]

**Supplemental Table S1 Multivariable logistic regression and trend analysis of LGBM model-predicted risk for HAP after adjustment for clinical covariates**

| **Exposure** | **Model 1 OR (95%CI) P-Value** | **Model 2 OR (95%CI) P-Value** | **Model 3 OR (95%CI) P-Value** |
| --- | --- | --- | --- |
| **LGBM model** | 134.588 (34.039, 532.151) <0.001 | 79.791 (18.457, 344.940) <0.001 | 10.237 (1.035, 101.223) 0.047 |
| **LGBM model group** |  |  |  |
| **0.157 - 0.262** | 1 | 1 | 1 |
| **0.263 - 0.333** | 1.775 (0.906, 3.477) 0.095 | 1.833 (0.905, 3.714) 0.093 | 2.548 (1.082, 5.999) 0.032 |
| **0.334 - 0.470** | 2.925 (1.526, 5.607) 0.001 | 2.892 (1.446, 5.784) 0.003 | 3.076 (1.279, 7.398) 0.012 |
| **0.478 - 0.956** | 10.862 (5.561, 21.216) <0.001 | 9.459 (4.601, 19.444) <0.001 | 6.316 (2.009, 19.861) 0.001 |
| **LGBM model**  **P for trend** | 341.020 (75.328, 1543.835) <0.001 | 227.673 (43.524, 1190.942) <0.001 | 46.704 (3.174, 687.113) 0.005 |

**Model 1: Non-adjusted.**

**Model 2: Adjust : Gender, Age, Hypertension (HP), :Diabetes Mellitus(DM), Smoking, Drinking.**

**Model 3: Adjust: Gender, Age, Hypertension (HP), :Diabetes Mellitus(DM), Smoking, Drinking, Glasgow Coma Scale(GCS), White Blood Cell count(WBC).**
